# Targeted Drug Delivery in Plants: Enzyme‐Responsive Lignin Nanocarriers for the Curative Treatment of the Worldwide Grapevine Trunk Disease Esca

**DOI:** 10.1002/advs.201802315

**Published:** 2019-05-29

**Authors:** Jochen Fischer, Sebastian J. Beckers, Doungporn Yiamsawas, Eckhard Thines, Katharina Landfester, Frederik R. Wurm

**Affiliations:** ^1^ IBWF gGmbH Institute for Biotechnology and Drug Research Erwin‐Schrödinger‐Str. 56 67663 Kaiserslautern Germany; ^2^ Max‐Planck‐Institut für Polymerforschung Ackermannweg 10 55128 Mainz Germany; ^3^ Microbiology and Wine Research at the Institute of Molecular Physiology (IMP) Johannes Gutenberg‐University Johann‐Joachim‐Becherweg 15 55128 Mainz Germany

**Keywords:** agriculture, grapevine trunk diseases, miniemulsion, nanocarriers, plant protection

## Abstract

Nanocarrier (NC)‐mediated drug delivery is widely researched in medicine but to date has not been used in agriculture. The first curative NC‐based treatment of the worldwide occurring grapevine trunk disease Esca, with more than 2 billion infected plants causing a loss yearly of $1.5 billion, is presented. To date, only repetitive spraying of fungicides is used to reduce chances of infection. This long‐term treatment against Esca uses minimal amounts of fungicide encapsulated in biobased and biodegradable lignin NCs. A single trunk injection of <10 mg fungicide results in curing of an infected plant. Only upon Esca infection, ligninolytic enzymes, secreted by the Esca‐associated fungi, degrade the lignin NC to release the fungicide. The specific antifungal activity is confirmed in vitro and in planta (in *Vitis vinifera* L. cv. ‘Portugieser’). All treated plants prove to exhibit significantly fewer symptoms several weeks after treatment, and their condition is monitored for 5 years (2014–2018), proving a long‐term curative effect of this NC treatment. This study proves the efficacy of this NC‐mediated drug delivery for agriculture, using a minimum amount of fungicides. It is believed that this concept can be extended to other plant diseases worldwide to reduce extensive spraying of agrochemicals.

## 1 Introduction

**Scheme 1:**
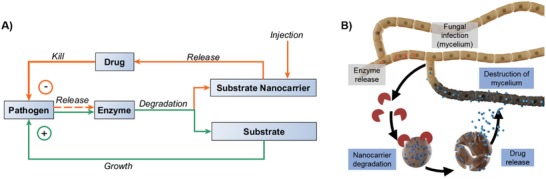
Concept of the NC‐mediated drug delivery: A) green pathway: degradation of a substrate leads to growth of the pathogen (infection, disease); orange pathway: a negative feedback loop is initiated by masking a drug that fights the pathogen in NCs based on the food source of the pathogen. B) Concept shown in (A) used to fight the fungal GTD “Esca:” fungicide‐loaded lignin NCs release the drug load only when the Esca fungi release lignin‐degrading enzymes.

The development of nanomaterials for targeted drug delivery with a release on demand is heavily researched in medicine.^1^ Such NCs are designed to respond on external stimuli at sites of infection, which trigger release of loaded therapeutics.^2^ As bacteria or fungi secrete certain enzymes (**Scheme** 1A, green pathway), a pathogen‐specific mechanism for drug release is available, relying on negative feedback, i.e., destruction of the pathogen, after enzymatic degradation of a nanocarrier (NC; Scheme 1A, orange pathway). If the NCs are composed of a nutrient for the pathogen, they will be consumed and degraded by the enzymes, which trigger “unintentionally” a drug release (Scheme 1A). This strategy releases a drug only after an infection and may act as a depot or vaccination for future infections. For the first time, this concept was demonstrated for a fungal disease in living plants, which were infected with the worldwide occurring disease “Esca” (Scheme 1B).

A conventional method to distribute herbicides and pesticides in agriculture is unspecific spraying and of 1–2.5 million metric tons of annually applied active ingredients only 30–40% reach targeted crops.^3^ This is because the sprayed agrochemicals are washed off by rainfall or are degraded by light, temperature, or microorganisms.^4^ Application of higher drug amounts and more frequent spraying could compensate for these effects, but would also increase costs and environmental contamination. Thus, to minimize the amounts of agrochemicals required, an efficient delivery method for plants is needed, which can withstand rain and guarantees durable protection after application.

Here, we present the first long‐term effective, curative NC‐mediated treatment against fungal trunk infections of grapevine plants. This disease is called Esca^5^ and is based on a series of fungal strains, which infect millions of grapevine plants worldwide every year.^6^, ^7^ As no curative treatment is available, the infected grapevines must be replaced, which leads to a financial loss of ≈$1.5 billion per year. Up to now, there has not been an effective curative treatment against Esca, resulting in drastic spreading of this disease around the world. Only preventive spraying of fungicides several times per year (up to 650 mg per year and plant) can reduce the danger of infection, which is detrimental for the environment and uneconomical.

Instead of extensive spraying, we provide a fungicide, encapsulated in a protective NC by a single injection into the trunk. Even with a minimum amount of less than 10 mg fungicide per plant, the disease symptoms were reduced significantly. Esca is associated with the occurrence of several fungi, including the tracheomycotic *Phaeomoniella chlamydospora* (Pch) and *Phaeoacremonium minimum* (Pmi).^4^, ^7^, ^8^ These pathogenic fungi segregate lignin‐degrading enzymes (e.g., laccases and peroxidases). Therefore, the substrate lignin was chosen as a carrier material, to develop an enzyme‐responsive drug delivery system. Since the disease‐associated fungi degrade the loaded lignin NCs, the fungicide is released exclusively in the case of infection, which allows not only a curative treatment but also long‐term protection (Scheme 1).

**Figure 1:**
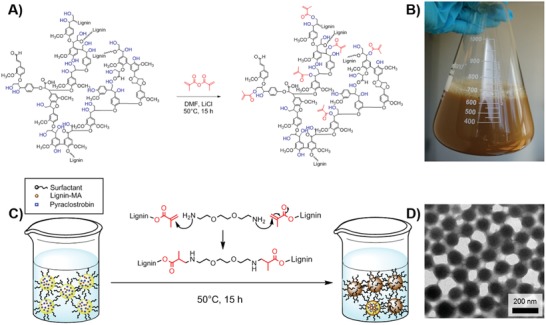
Synthesis of methacrylated‐lignin and formation of lignin NCs by crosslinking in miniemulsion. A) Methacrylation of lignin with methacrylic anhydride (note: representative structure of lignin may vary depending on the biological source). B) Photo of ≈600 mL of lignin NC dispersion (1 wt%). C) Crosslinking mechanism of lignin NCs in miniemulsion. D) Transmission electron microscopy image of lignin NCs.

The lignin NCs were prepared by crosslinking methacrylated Kraft lignin in the presence of a nonsystemic drug pyraclostrobin by a combination of miniemulsion polymerization and solvent evaporation.^9^ This procedure directly produces an aqueous dispersion of the drug‐loaded NCs and can be easily scaled‐up to several liters in the lab using standard equipment (**Figure** 1). Furthermore, no leakage of the drug was detected from the NCs during storage. The lignin NCs were studied in vitro and in planta against Esca pathogens: Only in the presence of lignin‐degrading fungi, pyraclostrobin was released and became active against the fungal infection. From a vineyard containing approximately 3000 plants, we selected 43 plants with early symptoms of Esca for the treatment. Injections of the NC dispersions into the trunks of these plants were conducted and the plants were monitored over a period of 5 years for effectiveness against Esca. In all treated plants, the Esca symptoms were reduced rapidly, and most treated plants were found with considerably less Esca symptoms than the control plants after 1 year. The majority of the treated plants did not show any signs of Esca during the following 3 years. This is the first demonstration treating Esca‐infected grapevine with a minimum amount of 10 mg fungicide and a single dose treatment. We believe that this general strategy of NC‐mediated drug delivery enabling a selective release by pathogen‐specific enzymes can be further extended to other plant diseases and will facilitate reducing the extensive amounts of agrochemicals distributed by conventional spraying.

## 2 Results and Discussion

*Synthesis of Lignin NCs*: In order to allow the encapsulation of the hydrophobic fungicide pyraclostrobin, we modified the hydroxyl groups of the hydrophilic Kraft lignin with methacrylic groups by the reaction with methacrylic anhydride (Figure 1A). Both NMR and IR spectroscopy proved the successful modification of lignin with methacrylic groups (Figure S3 in the Supporting Information: IR: ester bond vibrations at 1736 cm^−1^ (—C=O); reduction of the hydroxyl vibration (—OH) at 3000–3800 cm^−1^ after the reaction, indicating a high degree of functionalization). ^1^H NMR spectroscopy proved the attachment of the methacrylic group by the distinctive resonances of the vinyl group at 5.88 and 6.22 ppm. The diffusion‐ordered NMR spectroscopic ^1^H NMR analysis confirmed a covalent coupling of the vinyl groups to the polymeric lignin (Figure S4, Supporting Information), as the two protons of the vinyl group were detected at the same diffusion coefficient as the lignin backbone at approximately 1.7 × 10^10^ m^2^ s^−1^. The number of methacrylates was determined by the method of Balakshin and Capanema using 2‐chloro‐4,4,5,5‐tetramethyl‐1,3,2‐dioxaphospholane as a phosphorylation agent and found to be 5.5 mmol g^−1^ (Figures S5 and S6, Supporting Information).^10^

Lignin NCs were prepared by crosslinking the methacrylated lignin in miniemulsion droplets: methacrylated lignin was dissolved together with the fungicide pyraclostrobin in chloroform to generate the dispersed phase. The solution was then mixed with the aqueous phase containing either sodium dodecyl sulfate (“SDS,” an anionic surfactant) or Lutensol AT50 (“Lut,” a nonionic poly(ethylene glycol)‐based surfactant) as a stabilizer. A stable miniemulsion with homogenously distributed droplets was prepared by ultrasonication of the hydrophobic and the hydrophilic solutions. By using a microfluidizer instead of a sonication tip, the process was scaled easily to the liter‐level (Figure 1B). Afterward, 2,2'(ethylenedioxy)bis(ethylamine), which is soluble both in the aqueous and in the organic phase, was added to crosslink the methacrylated lignin via an aza‐Michael addition at 50 °C over a period of 15 h. Finally, chloroform was evaporated at room temperature to yield a stable lignin NC dispersion. Solid‐state ^13^C NMR spectroscopy of the freeze‐dried product proved the covalent crosslinking of the NCs (Figure S7, Supporting Information).

Depending on the preparation conditions, NCs with diameters between ≈200 and 700 nm (determined by dynamic light scattering, DLS) were obtained. The sizes of the NCs were varied by adjusting the concentration of lignin‐MA in the droplets and the amount and type of surfactant (Table S1, Supporting Information). The addition of pyraclostrobin to the miniemulsion did not result in any significant change of the NC size, compared to “empty” lignin NCs. Transmission electron images show the spherical morphology of all fungicide‐loaded lignin NCs (Figure 1D). The diameters of the NCs were monitored over a period of several months at room temperature but did not significantly change, proving high colloidal stability without aggregation or degradation during storage. Furthermore, the encapsulation efficiency for pyraclostrobin was determined by high‐performance liquid chromatography (HPLC) and found to be typically above 90%. Even at 20 wt% of fungicide loading, an encapsulation efficiency of more than 97% was obtained (Table S1, Supporting Information). Besides, no pyraclostrobin was released from the lignin NC during storage over a period of at least 1 month proving that no cargo is lost by leaking.

**Figure 2:**
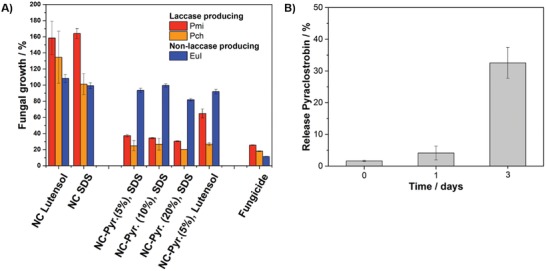
In vitro investigations of lignin NCs. Pch and Pmi were used as ligninolytic enzyme‐segregating fungi, whereas Eul was applied as control fungus without laccase segregation. A) Optical density (at 600 nm) of fungal cultures in 96‐well microtiter plates after 48 h for samples empty and pyraclostrobin‐loaded lignin NCs (see Table S1 for details, Supporting Information). B) HPLC‐MS analysis of NC supernatant after the incubation with culture filtrate of Pch after 24 and 72 h. Release compared to a pyraclostrobin standard curve and the amount of applied fungicide.

*In Vitro and In Vivo Antifungal Activity*: The drug release of lignin NCs with a pyraclostrobin loading of 20 wt% was quantified in vitro by HPLC‐MS (mass spectrometry). After mixing an NC dispersion with the filtrate of a 72 h well‐grown culture from the Esca‐associated fungus Pch, more than a third of the encapsulated fungicide was released to the medium, proving that the segregated lignolytic enzymes can induce the degradation of the NC matrix (**Figure** 2B). A complete drug release could not be measured as the released drug effectively destroyed the fungi during the experiment. In previous studies, we already used isolated enzymes to degrade lignin NCs in vitro, but no living fungi were involved so far.^11^ Furthermore, those results indicate that a drug release in planta is not affected by xylem sap, since a similar pH level was used for the test compared to plant sap.^12^ In addition, aggregation tests in grapevine canes were conducted and no influencing effects to lignin NCs activity were observed. In further tests, the drug release was analyzed by determining the growth‐inhibition of spores and mycelium belonging to the Esca‐associated fungi Pch and Pmi in a malt yeast medium supplemented with lignin NCs. To prove the selective enzymatic release of pyraclostrobin, *Eutypa lata* (Eul), which does not produce lignin‐degrading enzymes, was used as a control fungus.^13^ Eul grew unaffected in the presence of the drug‐loaded NCs, proving no release of the fungicide and thus the density of the NCs without the presence of lignin‐degrading enzymes (Figure 2A, blue bars).

Pch and Pmi, however, which produce lignin‐degrading enzymes, were able to release the drug and their growth was suppressed completely. An additional proof that the lignin NCs act as nutrient for these fungi is obvious, when “empty” NCs were incubated with Pch and Pmi: the fungal growth was even higher after the addition of the NCs without encapsulated fungicide (Figure 2A, “NC SDS” and “NC Lutensol”) than in the negative control (medium only). Both SDS and Lutensol did not influence the mycelium growth and the lignin carrier material itself may have acted as a nutrition source. We could, therefore, prove that the NCs alone or the surfactants did not inhibit fungal growth. In contrast, all fungicide‐loaded NCs resulted in the suppression of growth of Pch and Pmi. NCs with different drug loading concentrations (5, 10, and 20 wt% pyraclostrobin compared to lignin), surfactant, or diameters exhibited similar activities against the fungi (Figure 2A). Importantly, the results prove that the lignin NCs (NC‐Pyr. (20%), Sample 4, Table S1, Supporting Information) stabilized with SDS exhibit almost the same germination inhibiting rate as the positive control. Furthermore, we observed that lignin‐NC dispersions stored at 4 °C for over 2 years remained the same antifungal activity, proving their long shelf‐life.

Whereas the NCs should kill the Esca fungi, they should not harm plant cells in general. To elucidate the phytotoxicity of the lignin NCs on plant cells, the dispersions were tested on *Vitis* callus cultures (DSMZ‐ PC‐1137). No effect on the viability of the callus cells in the presence of the lignin NCs was observed (Figure S1B summarizes the data; Figure S1A shows a microscopy image of the callus cultures, Supporting Information). In contrast, a positive control (the herbicide glufosinate‐ammonium) causes the cell death of the *Vitis* callus cells. These experiments prove that the lignin NCs (loaded with the fungicide pyraclostrobin and “empty”) are nonphytotoxic to plant cells, making them attractive candidates to treat various plant diseases.

**Figure 3:**
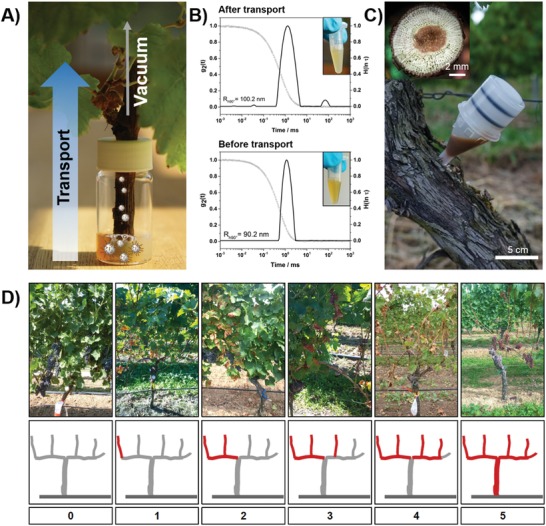
A) Shoot of a young grapevine plant through which a NC dispersion was sucked by vacuum simulating the natural transpiration pull. B) Photos and respective particle size distributions of the lignin NC dispersion before and after transport through a grapevine shoot. C) Injector filled with 5 mL of 1 wt% lignin NC dispersion and cross‐section of a grapevine plant, showing the size of the vascular bundles. D) Categorization of Esca symptoms determined in field trials graduated in six severity degrees (gray: normal growth, red: Esca symptoms observable).

*In Planta Studies*: Up to now, there are only two injection‐based drug delivery systems for plant protection: i) injection of soluble systemic insecticides in loblolly pine trees against southern pine bark beetles.^14^ ii) Injection of fungicides in trees against apple scab (*Venturia inaequalis*) and powdery mildew (*Phyllactinia sp*.) was able to reduce disease severity.^15^ Such strategies, however, rely on a systemic treatment of a plant with the drug. To date, no nano‐ or microcarriers were able to protect plants from diseases and release the cargo only in case of infection. Thus, the colloidal properties of the NCs inside of the plant are unknown. Do they aggregate, when in contact with the fluid in phloem or xylem? Or do they retain their colloidal stability and can be transported inside of the plant? To study the colloidal stability, which is required for a potential transport of the NCs inside of living plants, we used DLS in “wood extract” (see the Supporting Information for details). This complex mixture simulates the conditions inside of a plant. DLS was used to assess the colloidal properties, following a method established by Rausch et al. for the determination of NC aggregation in human blood plasma.^16^ After the addition of the NC dispersion to the “wood extract,” no macroscopic phase separation occurred and DLS proved only a few smaller aggregates <1 µm, indicating a potential transport inside of the plant tissue. This was further supported by an in planta experiment, in which we passed an NC dispersion through a shoot of a young grapevine plant (**Figure** 3A). A vacuum of 75 mbar was applied, simulating the natural transpiration pull.^17^ Also in this experiment, the size distribution determined by DLS remained almost unchanged after the transport through the vascular plant tissue (Figure 3A,B). These experiments indicate the colloidal stability and therefore the ability for transport inside of the plant. In ongoing research, the NC transport inside of living plants is studied and will be reported in near future.

After the verification that the lignin NCs can distribute within the plant through the vascular tissue as well as their antifungal activity and nonphytotoxicity in vitro, the aqueous dispersions were injected in planta into *Vitis vinifera* L. cv. ‘Portugieser’ plants in a vineyard in Forst an der Weinstraße, Germany. An early symptom of Esca is necrotic yellow‐brownish tissues around the leaf veins described as “tiger stripes” (see images in Figure 3D).^18^ Often accompanied by wilting grapes, showing brown spots on the surface and becoming inedible.^19^ Cross‐sections of infected trunks show necrotic tissue with brown to black spots and dry wood (Figure S10, Supporting Information).^20^ We categorized the progress of Esca symptoms in six categories (Figure 3D). The plants were cultivated with two branches, which allowed a ranking system based on the number of affected twigs of each plant. All selected grapevine plants showed symptoms of an early stage of an Esca infection (≈1–2 on the severity level).

**Figure 4:**
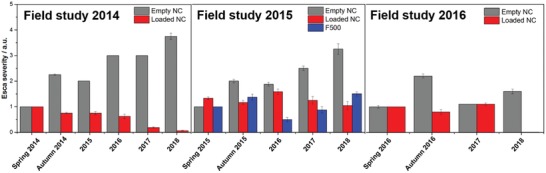
In planta application of lignin NCs. Boxplot showing severity of wine plants in the years 2015 until 2018: comparison between treatments with empty lignin NCs (i.e., without loaded fungicide); lignin NCs (loaded with pyraclostrobin (0.7 mg mL^−1^)); F500 (i.e., the injection of the F500 formulation (BASF) with pyraclostrobin (6 mg mL^−1^)). (Note: field study started in 2016: all plants treated with the loaded NCs did not show any signs of Esca in 2018).

Into the plant's trunk, a hole was drilled (6 mm diameter, 8 mm depth) and the aqueous NC dispersion was injected (≈1 wt% solid content) via a microinjection system (from TreeTec, Morriston, Florida) over a period of several hours (Figure 3C). Afterward, the wound was sealed with a pruning wound seal product. In total, 70 plants were treated with different NC dispersions. In addition, all 2958 plants of the vineyard were monitored over a period of 5 years (2014–2018). Forty‐three plants were treated with the pyraclostrobin‐loaded lignin NCs, 19 plants were treated with “empty” lignin NCs, 8 plants were treated by the injection of an F500 formulation (commercialized product of BASF, Germany), and the remaining 2877 plants were not treated. The Esca symptoms on all plants untreated or treated with “empty” lignin NCs (see **Figure** 4 “empty NC,” gray) increased significantly during the next months and more particularly the following years. After 12 months, these plants either did not survive or showed more severe Esca symptoms. In contrast, the fungicide‐treated plants (0.7 mg mL^−1^ encapsulated pyraclostrobin) showed a significant improvement in their conditions after 3 months and up to 4 years. In the first year, the overall Esca severity was reduced <1 (Figure 4, field study 2014) and plants treated with loaded lignin NCs showed 62% (Autumn 2014) and 58% (after 2 years, 2016) fewer symptoms than the control plants (Figure S9 in the Supporting Information shows representative photographs of a treated and a nontreated plant from 2014 to 2018). Furthermore, monitoring of the treated plants in the following 2 to 4 years, respectively, proved a further improvement as less or even no leaf symptoms (in 2018) were detectable. Two additional field trials were started in 2015 and 2016: in 2015, we compared the NC treatment with a trunk injection of the commercial formulation (F500, 6 mg mL^−1^ pyraclostrobin). Treatment with F500 resulted in an initial improvement (2016) of the symptoms (Figure 4, field study initiated in 2015), but in the following years (2017 and 2018) the symptom level increased again, although an almost nine higher amount of the fungicide was injected in the commercial suspension. In contrast, the symptoms of the NC‐treated plants further decreased over the following years, even without additional injections. An additional field study was started in 2016, and again after 2 years, no leaf symptoms were visible in 2018, while the nontreated plants still showed Esca symptoms. These results clearly prove the release of the fungicide and the long‐term curative effect of the treatment (*note*: the plants are still under observation for the next years).

To our knowledge, the herein reported lignin NCs is the first report of an injection system based on long‐term active and stimuli‐responsive NCs for drug delivery inside of living plants. In addition, these results prove that lignin NCs, loaded with a hydrophobic fungicide and injected into the trunk of wine plants, act as a curative treatment for Esca‐infected grapevine plants. Together with the in vitro studies, we also suppose no release of the fungicide in a noninfected plant, as only the laccase segregated from the fungi, would open the NCs. The lignin NCs are an effective cure to treat already Esca‐infected grapevine plants. All plant protection product treatments that are currently available in the market, only aim a preventive fungal infection, but are not custom made for GTD (grapevine trunk disease) pathogens or other trunk diseases, e.g., in plums, peaches, or almonds and are futile after the infection and colonization in the plant occurred. Since GTD pathogens grow mostly in the xylem vessels, we assume that the introduced injection method is capable to reach the fungal mycelium inside the plant.^21^

**Figure 5:**
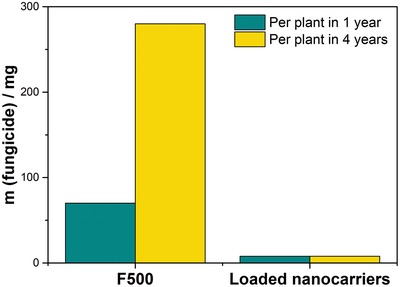
Comparison of fungicide amounts used via the trunk injection of the lignin NCs (right) compared to preventive spraying of F500 (left) in 1 year (green) or over a period of 4 years (yellow) according to ref. 22.

Another important advantage of the proposed method is the significant reduction of the amount of fungicide applied per plant. This drastically reduces the release of fungicide to the ecosystem. The amount recommended for one foliar application in conventional viticultural farming of a strobilurine derivative in a German vineyard is at least 70 mg (after blossom) but up to 640 mg (at full foliation) per year and plant depending on the month of spraying.^22^ In contrast, by a single injection of the enzyme‐responsive lignin NCs into the trunk, we are able to reduce the amount of fungicide to less than 3% compared to preventive spraying without the need for treatment in the following years (**Figure** 5). Furthermore, handling of the aqueous NC dispersion is safe and easy and prevents unwanted release of the fungicide into the environment as the fungicide is embedded into the plant and not sprayed unselectively, without the undesired release of the drug.

Another important finding was that after the treatment with the lignin NCs no pyraclostrobin was found in the harvested grapes (≈0.5 kg were analyzed, compare Supporting Information). In contrast to the injected NCs, it was reported that sprayed fungicides remain in/on the grapes after harvesting: e.g., Angioni et al. proved the presence of boscalid in grapes and wine.^23^ While the maximum residue limit by the EU commission was not reached, still the amount of up to 4.23 mg kg^−1^ boscalid was detected in the samples. Also, other fungicides such as iprovalicarb and indoxacarb were detected in the same samples. In another test series 1.01 mg kg^−1^ of iprodione; 0.78 mg kg^−1^ of procymidone; and 0.37 mg kg^−1^ of vinclozolin were detected in grapes.^24^ These results further underline that fungicides applied by conventional spraying are always traceable in grapes, whereas drug delivery via the lignin NCs enables a trace‐free harvest already in the first year after the application.

## 3 Conclusion

In conclusion, this work presents a general approach for NC‐mediated drug delivery in living plants. Although using minimal amounts of fungicide compared to a preventive spraying strategy, this concept successfully acted as the first curative treatment for a GTD “Esca.” With lignin as the matrix of the NCs, the Esca‐associated fungi degraded the trunk of the grapevine plant and the NC sufficiently, which results in an enzyme‐responsive drug release. These NCs, loaded with pyraclostrobin, were prepared via a miniemulsion polymerization, providing an aqueous NC dispersion with adjustable drug loading and high encapsulation efficiency. The enzyme‐triggered fungicide release was demonstrated by in vitro experiments. Thereafter, we injected this aqueous dispersion into the trunks of infected and symptomatic grapevine plants using a ready‐made injection system. The NCs were nonphytotoxic and do not release the drug unless an Esca‐associated pathogen (Pmi and Pch) secretes lignin‐degrading enzymes. Furthermore, a single trunk injection resulted in a significant reduction of Esca symptoms monitored in plants over a period of 5 years. This concept provides as curative treatment against Esca, but might be extended as a preventive treatment as no drug is released without an Esca infection. Hence, by transferring the controlled drug delivery approach from medicine to agriculture, we envision establishing an inexpensive and targeted technique for advanced plant protection. This general strategy will be extended for various plant diseases such as plum and peach infections and will contribute to decreasing untoward environmental effects from pollution of agrochemicals.

## Conflict of Interest

The authors declare no conflict of interest.

## Supporting information

Supplementary
