# Supplementary material for: Targeted Drug Delivery in Plants: Enzyme‐Responsive Lignin Nanocarriers for the Curative Treatment of the Worldwide Grapevine Trunk Disease Esca
Source: Adv Sci (Weinh). 2019 May 29;6(15):1802315. doi: 10.1002/advs.201802315 (PMC6685467; doi:10.1002/advs.201802315)
Supplement: Supplementary file 1 — Supplementary [file ADVS-6-1802315-s001.pdf]

Copyright WILEY-VCH Verlag GmbH & Co. KGaA, 69469 Weinheim, Germany, 2019.

ADVANCED SCIENCE
Open Access

Supporting Information

for *Adv. Sci.,* DOI: 10.1002/advs.201802315

Targeted Drug Delivery in Plants: Enzyme-Responsive Lignin Nanocarriers for the Curative Treatment of the Worldwide Grapevine Trunk Disease Esca

Jochen Fischer, Sebastian J. Beckers, Doungporn Yiamsawas, Eckhard Thines, Katharina Landfester, and Frederik R. Wurm**

Supporting Information

Targeted Drug Delivery in Plants: Enzyme-Responsive Lignin Nanocarriers for the Curative Treatment of the Worldwide Grapevine Trunk Disease Esca

Jochen Fischer[a,*], Sebastian J. Beckers[b], Doungporn Yiamsawas[b], Eckhard Thines[a,c], Katharina Landfester[b], and Frederik R. Wurm[b,*]

[a] *IBWF gGmbH, Institute of Biotechnology and Drug Research, Erwin-Schrödinger-Str. 56, 67663 Kaiserslautern, Germany. fischer@ibwf.de*

[b] *Max-Planck-Institut für Polymerforschung, Ackermannweg 10, 55128 Mainz, Germany, wurm@mpip-mainz.mpg.de*

[c] *Institute of Molecular Physiology (IMP), Johann-Joachim-Becherweg 15, Johannes Gutenberg-University, 55128 Mainz, Germany.*

Syntheses:

Materials

Kraft lignin (alkaline, 95%), lithium chloride (99%), triethylamine (99%), methacrylic anhydride (94%), sodiumdodecylsulfate (99%), 2,2′-(ethylenedioxy)bis(ethylamine) (98%) and endo-N-hydroxy-5-norbornene-2,3-dicarboximide (97%) were purchased from Sigma Aldrich. The solvents dimethylformamide and chloroform were of analytical grade and supplied from Fischer Chemicals. Pyraclostrobin (98%) and hexamethylcyclotrisiloxane (98%) were bought from Toronto Research Chemicals and Acros. Lutensol AT50 was provided by the BASF.

Synthesis of methacrylated lignin

2 g of lignin (12.26 mmol OH groups) was added to 60 ml LiCl/dimethyl formamide (DMF) and heated under an argon atmosphere to 90 °C until completely dissolved. After cooling to 50 °C, 1 ml of triethylamine (10 mmol) was added and the mixture was stirred for further 15 min. 3 ml of methacrylic anhydride (20 mmol) was then injected over 30 min and the reaction was allowed to proceed at 50 °C overnight. The mixture was precipitated into a large excess of isopropyl alcohol and isolated by centrifugation. When precipitated further three times, the product was dried at room temperature under vacuum. Typically yields of 60% were obtained. By ^{1}H and ^{31}P-NMR spectroscopy, a hydroxyl group conversion of 90% (5.5 mmol/g methacrylic groups) was determined.

Preparation of crosslinked-lignin nanocarriers

Crosslinked-lignin nanocarriers were prepared by a combination of miniemulsion polymerization and solvent evaporation. Typically, 500 mg (2.75 mmol methacrylic groups) of methacrylated-lignin and 100 mg (for a pyraclostrobin: lignin ratio of 20%) pyraclostrobin were dissolved in 5 g of chloroform ($CHCl_3$). In order to form a pre-emulsion, the mixture was given to 45 g of a surfactant solution (for concentrations see table 1) and stirred at 1000

rpm for 1 h. The emulsion was sonicated in the next step for 3 min (1/2 inch tip, 70 % amplitude, 20 s ultrasound followed by 10 s pause) under ice cooling to prevent any solvent evaporation. Afterwards, a solution of 250 mg 2,2´(ethylenedioxy)bis(ethylamine) in 5 mL water was added dropwise to the emulsion. The reaction was performed at 50 °C for 15 h. Finally, the solvent was evaporated by stirring the opened reaction vessel overnight at room temperature. The volume of the dispersion was adjusted to 50 mL by addition of water (this corresponds to a solid content of 19 mg/mL and a pyraclostrobin concentration of 2 mg/mL). The dispersion can be diluted to the desired concentration and is stable against coalescence or aggregation upon the addition of water. The nanocarriers obtained were characterized by dynamic light scattering (DLS) and transmission electron microscopy (TEM).

Table S1. Characterization data of lignin nanocarriers.

No.	surfactant type/	Surfactant [1] / mg/mL	Diameter [2]/ nm	PDI	pyraclostrobin: lignin mass ratio / %	encapsulation efficiency / %[3]
**1**	SDS	1.0	260	0.3	-	-
**2**	SDS	1.0	310	0.2	5	94
**3**	SDS	1.0	330	0.3	10	94
**4**	SDS	1.0	310	0.4	20	98
**5**	SDS	0.5	510	0.4	5	99
**6**	SDS	2.0	220	0.3	5	64
**7**	SDS	2.0	213	0.4	20	97
**8**	Lutensol AT50	1.0	640	0.3	5	100

1: surfactant concentration for nanocarrier synthesis.

2: determined by DLS.

3: detmined by HPLC.

Lignin modification: Characterization

In order to confirm the successful methacrylation of lignin, Fourier-transformed infrared (FT-IR) spectra were measured before and after functionalization using a Nicolet iS10 with Vertical ATR Accessory. Spectra were recorded from dried nanocarrier samples between 600 and 4000 cm^{-1}.

^{1}H and ^{31}P NMR (nuclear magnetic resonance) spectroscopy were performed on a Bruker AVANCE at 500 MHz using hexamethylcyclo trisiloxane as an internal standard. The samples for the ^{31}P-NMR spectra were prepared following the method of Balakshin *et al*. Herein, lignin's hydroxyl groups are first fully converted with 2-chloro-4,4,5,5-tetramethyl-1,3,2-dioxaphospholane and quantified afterward using the phosphorylated endo-N-hydroxy-5-norbornene-2,3-dicarboximide as an internal standard (Figure S3).[1] Lignin's hydroxyl groups are calculated as follows:

$$c(OH - Lignin) = \frac{n(Std.) \cdot Integral(150 - 137\,ppm)}{m(Lignin)} \left[\frac{mmol}{g}\right]$$

Lignin Nanocarriers: Characterizations

The hydrodynamic diameters of the particles were measured by a NICOMP 380 at a fixed angle of 90° and a laser diode running at 635 nm. The morphology of the particles was observed by transmission electron microscopy (TEM) using a Joel 1400 at an accelerating voltage of 120 kV. Samples were prepared by casting a drop of diluted dispersion on a carbon-coated copper grid (300 square meshes) and then dried at room temperature.

Solid State NMR

Solid State ^{13}C CP/MAS NMR spectra were obtained on a Bruker Avance II operating at 300MHz proton Larmor frequency equipped with a 4 mm MAS double resonance probe. All experiments were carried out at room temperature with MAS at 10 kHz. Peaks were referenced to alanine (177.9 ppm). A Contact times 3 ms were used.

Encapsulation Efficiency

In order to determine the encapsulation efficiency, the amount of pyraclostrobin loaded into the lignin nanocarriers was measured by high-pressure liquid chromatography (HPLC). Two methods were applied: (1) Indirect method: The sample was centrifuged at 10000 rpm for 60 min and the supernatant containing the non-encapsulated fungicide was freeze dried. Pyraclostrobin left was then dissolved in THF and quantified by HPLC. (2) Direct method: After isolation of the lignin-nanocarriers by centrifugation at 10000 rpm, the pellet obtained was freeze-dried and suspended in THF in order to extract the previously loaded drug. All solutions were passed through a 0.2 μm filter and analyzed by HPLC (Agilent Elicpse Plus RP18) using THF: water/ 0.1 %wt as mobile phase and TFA-gradient, injection volume was 10 μL and the column temperature maintained at 20ºC. The analysis was performed at a flow rate of 0.2 $mL \cdot min^{-1}$ with the UV detector at 280 nm. The encapsulation efficiency (EE) and the weight percentage of pyraclostrobin n nanocarriers were determined according to the following equations:

$$EE = \frac{\mathrm{m(pyraclostrobin\ in\ nanocarrier)}}{\mathrm{m(pyraclostrobin\ initial)}} \cdot 100\%$$

In vitro phytotoxicity test with Vitis vinifera *callus culture*

The callus culture (*Vitis vinifera* L. PC-1137, DSMZ) was incubated in 50 mL B5VIT medium for 6 days and then centrifuged (5 min, 1500 rpm, 22 °C) and dissolved in 45 mL of fresh B5VIT medium. 200µL of the cell suspension was incubated (40 rpm, 27°C) in a 96-well plate supplemented with 1µg of Pyraclostrobin-loaded nanocarriers. 20µL of the culture were stained with 2µL fluorescein-diacetate solution (1mg/10mL) after 24 hours to determine the number of viable cells (Widholm, 1972).

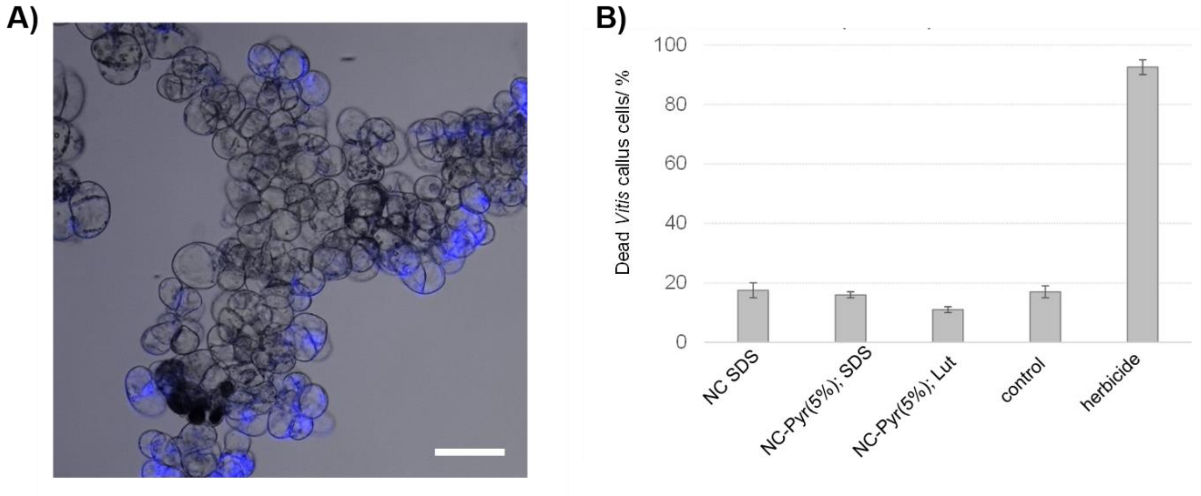

Figure S1. A) Fluorescent microscope image (Zeiss Axio Imager) scale bar = 100 µm; fluorescein diacetate (FDA) staining and artificial coloration (fluorescence filter 38; BP 525/50). B) Number of dead *Vitis vinifera* callus culture cells after 24 h (Samples: NC Lut/ SDS: lignin nanocarriers without fungicide; 2. NC-Pyr(x%): lignin nanocarriers loaded with different amounts of pyraclostrobin; SDS or Lut indicates the surfactant used for stabilization of the nanocarrier dispersion; controls: without the addition of nanocarriers or fungicide; herbicide: glufosinate-ammonium (0.5 mg/mL), fungicide: pyraclostrobin (0.5 mg/mL).

In vitro and in vivo antifungal investigations

Germination assay

Conidia from 18-day-old agar plate cultures were harvested. After centrifugation at 4000 rpm for 10 minutes, the conidia were re-suspended in HMG-medium (10 $g{\cdot}L^{-1}$ malt extract, 10 $g{\cdot}L^{-1}$ glucose, 4 $g{\cdot}L^{-1}$ yeast extract, pH 5.5) to a concentration of $1x10^5$ per mL. The test was carried out in 96-well microliter plates (Greiner Bio-One GmbH, Frickenhausen) with conidia in 200 μL of YMG-medium and an incubation time of 72 hours at 27 °C.[2] The optical density was measured at a wavelength of 600 nm (Benchmark Plus Microplate reader, BioRad, Munich). Tests were conducted in triplicates. The concentration of the used fungicide was 5 μg per 200 μL medium in a well.

Trunk injections:

The site of the trunk that was selected for the injection was sprayed with 5% sodium hypochlorite to sterilize the wooden surface of the plant. Afterwards, a ca. 8 mm deep and 6 mm wide hole was drilled into the grapevine trunk. The chosen plants had trunk diameters of at least 20 mm. The drill head was sterilized in 70% ethanol for 1 minute before the next plant was treated. After the drilling, the injection system (supplied from Tree Tech Microinjection Systems (FL, US), Figure S2A) was filled with 5 mL of the lignin nanocarrier dispersion and inserted to the grapevine trunk hole. The scroll spring pressure system was pushed after the injector was placed safely in the runk and the dispersion was allowed to flow into the trunk. After 24 h, the injection system was removed and the wound was sealed with grafting wax. The injector and the process of the application is presented in Figure S2.

The plants were monitored for five months after the injection. The monitoring ended when the autumn leaf coloration started. The plants were controlled on a six weeks' basis for increasing or decreasing foliar Esca symptoms in comparison to their appearance to the previous control and the untreated plants. The discolorations were documented using a digital camera. The plants were monitored in the following years for Esca leaf symptoms.

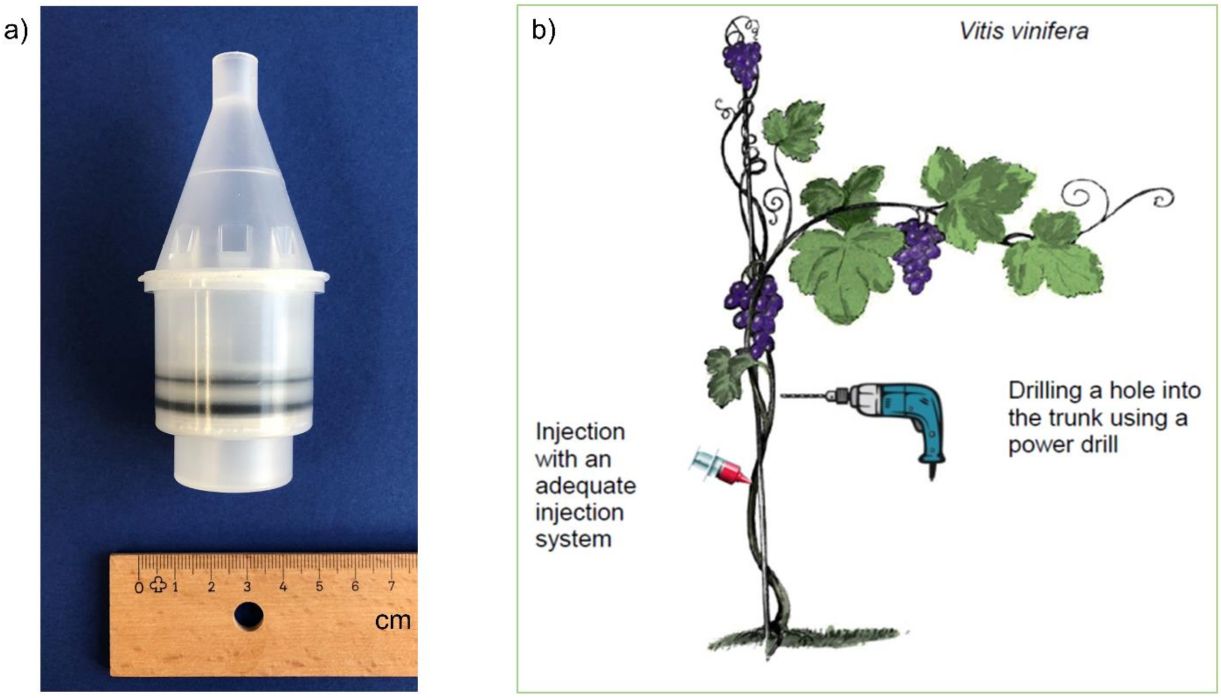

Figure S2. A) Injection device used for the in planta studies. B) Lignin-Nanocarrier injections procedure scheme; 1) drilling of the application inlet, 2) injection of the nanocarrier suspension.

Pesticide screening

Ca. 3 month after injection ca. 400 g of grapes were collected and analyzed on their pesticide content by LC-MS/MS following the method DIN EN 15662:2009-02, mod., P-14.141, LC-MS/MS. Eurofins / Dr. Specht Laboratorien GmbH (Hamburg; Germany) performed the screening. A pyraclostrobin content of < 0.01 mg/kg was detected.

Preparation of "wood extract"

20 g of lyophilized wood chips (length: 1-3 cm) of *Vitis vinifera* (18 years, from Rheinhessen, Germany) was blended for 1 min with 100 mL of water. Afterwards, the mixture was filtered through a paper filter to separate the solid. Prior to use, the extract was filter through a 5 μm syringe filter.

The wood extract is a mixture of xylem and phloem sap and has a pH of 6.6. According to literature, it contains a variety of sugars (mostly sucrose 100-300 g L^{-1}), amino acids (mostly glutamine 5-40 g L^{-1}), organic acids (mostly malate g L^{-1}) and inorganic ions.[3]

Colloidal stability in wood extract and after transport through a young grapevine plant.

A mixture containing 250 μL of a SDS stabilized lignin nanocarrier dispersion (1 wt%) and 750 μL of "wood extract" was kept for 48 h at room temperature. Before the light scattering experiments, 20 μL of the mixture was added to 1 mL of water and filtered through a 5 μm syringe filter. Dynamic light scattering (DLS) measurements were performed on a commercially available instrument from ALV GmbH, Germany consisting of an electronically controlled goniometer and an ALV-5000 multiple tau full-digital correlator. A He Ne laser with a wavelength of 632.8 nm and output power of 25 mW (JDS Uniphase, USA, Type 1145P) was utilized as the light source. The evaluation of the data was performed following the procedure of Rausch et al.[4]

Additional Spectra & Images:

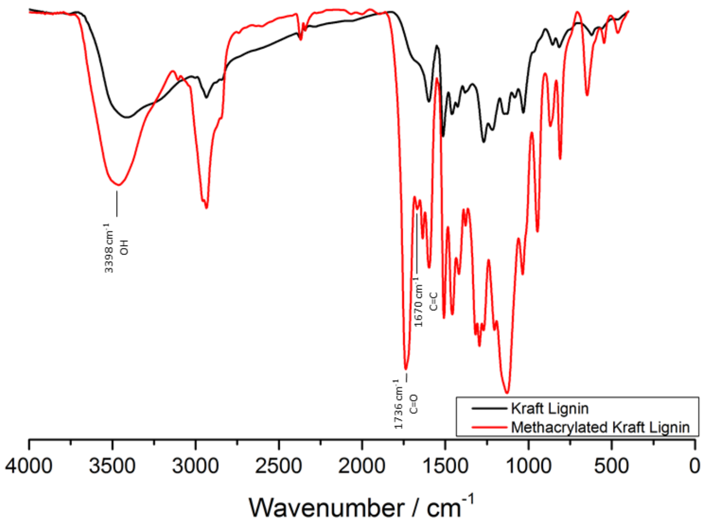

Figure S3. FT-IR spectra of Kraft lignin and methacrylated Kraft lignin.

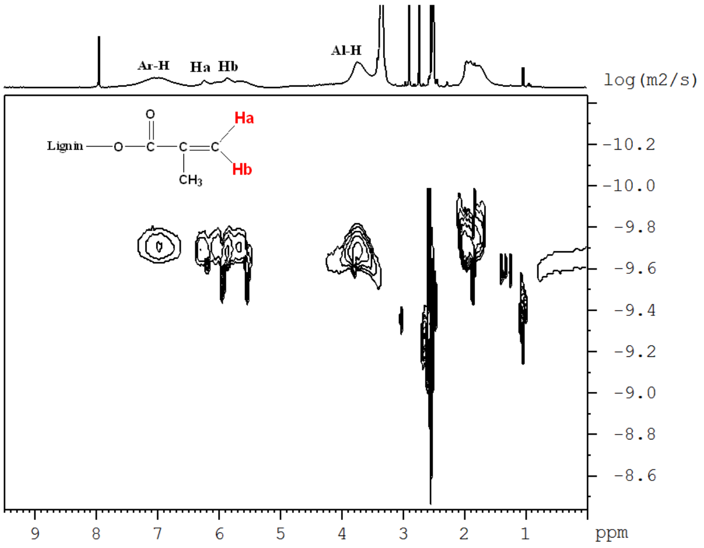

Figure S4. ^{1}H DOSY-NMR (700 MHz, 298 K, $CDCl_3$) spectra of methacrylated Kraft lignin.

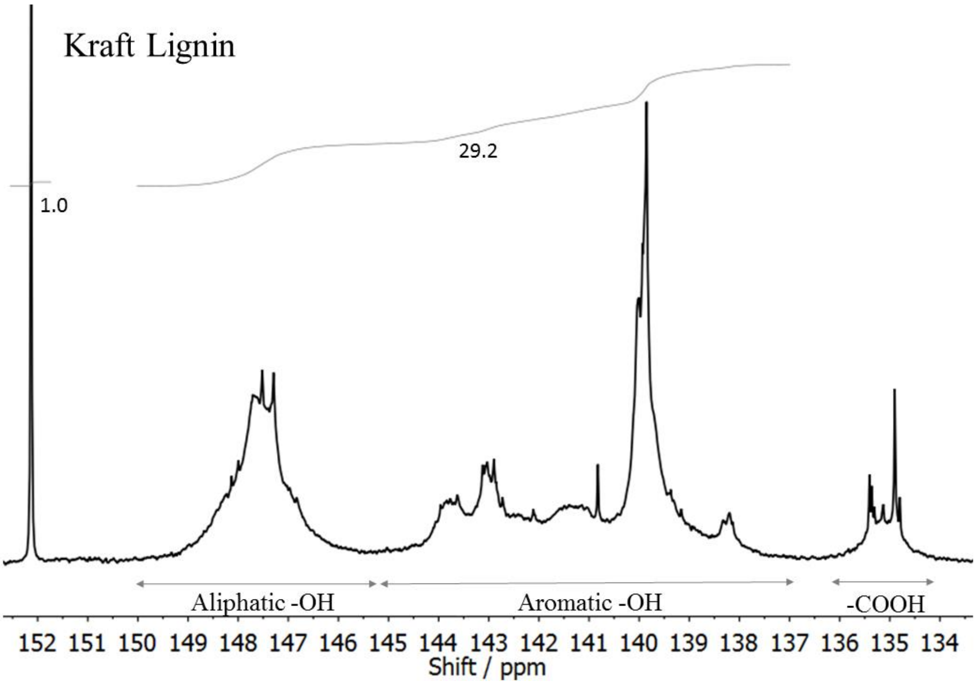

Figure S5. ^{31}P-NMR of Kraft lignin after reaction with 2-Chloro-4,4,5,5-tetramethyl-1,3,2-dioxaphospholane

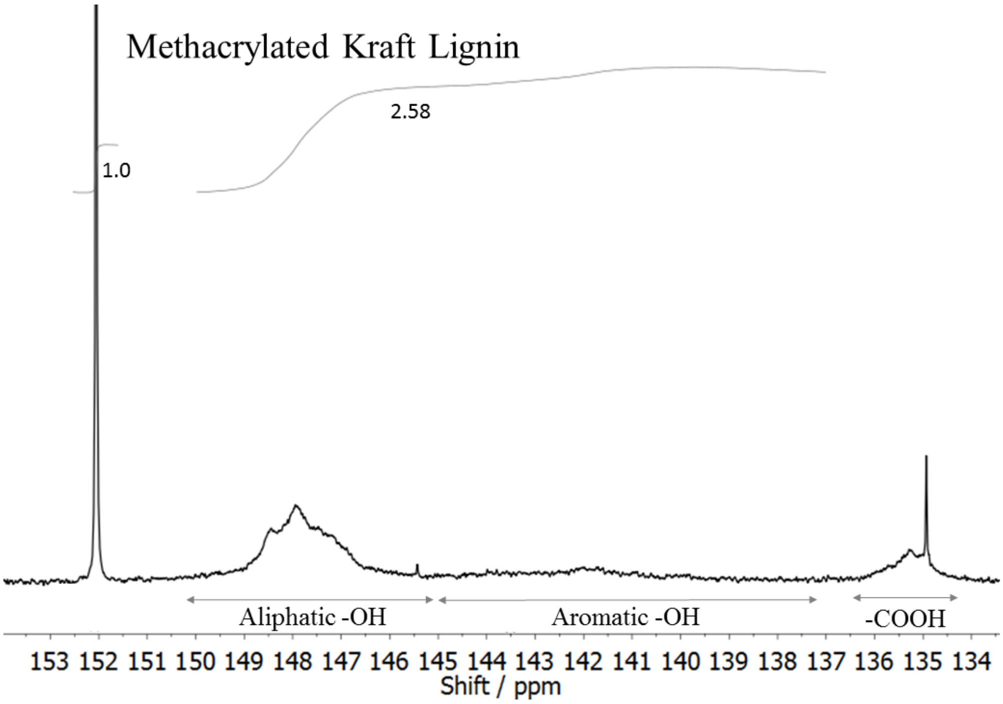

Figure S6. ^{31}P-NMR of methacrylated Kraft lignin after reaction with 2-Chloro-4,4,5,5-tetramethyl-1,3,2-dioxaphospholane.

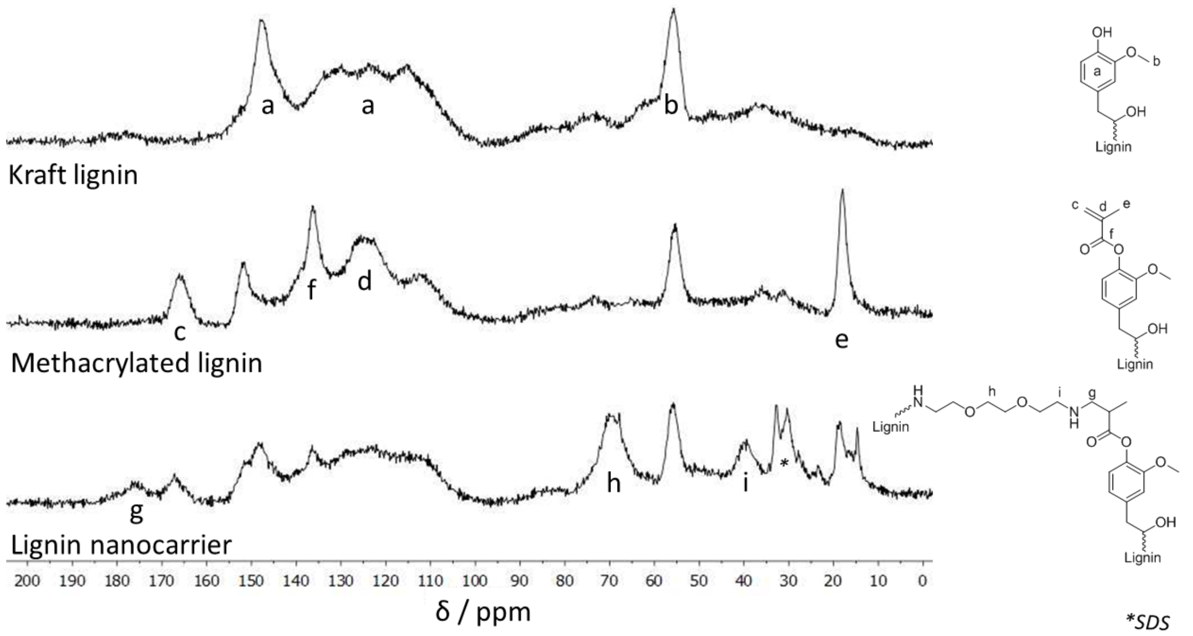

Figure S7. Solid State ^{13}C NMR spectra of Kraft lignin (top), methacrylated lignin (middle), and lignin nanocarriers (after drying of the dispersion, bottom) with peak assignments.

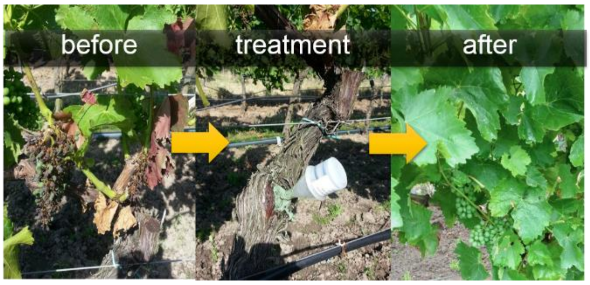

Figure S8: Photos on an Esca-infected grapevine plant (left), during treatment (middle), and after successful treatment (right).

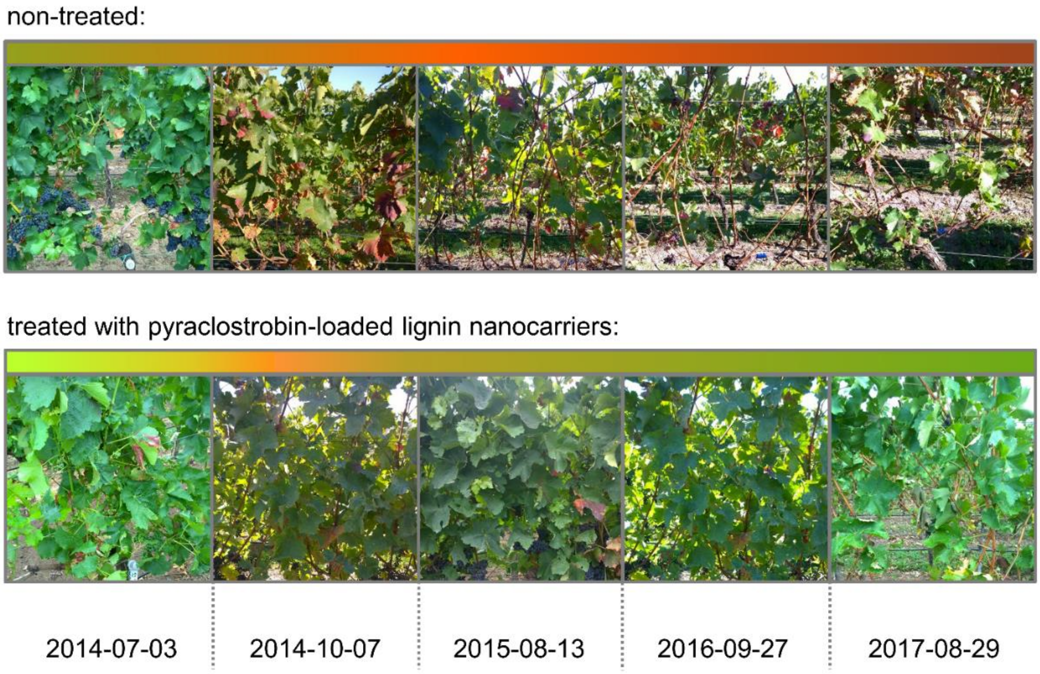

Figure S9. Photographs of a treated grapevine plant (with pyraclostrobin-loaded nanocarriers, top) and a non-treated plant (bottom) over a period of 4 years (dates are noted below the photos).

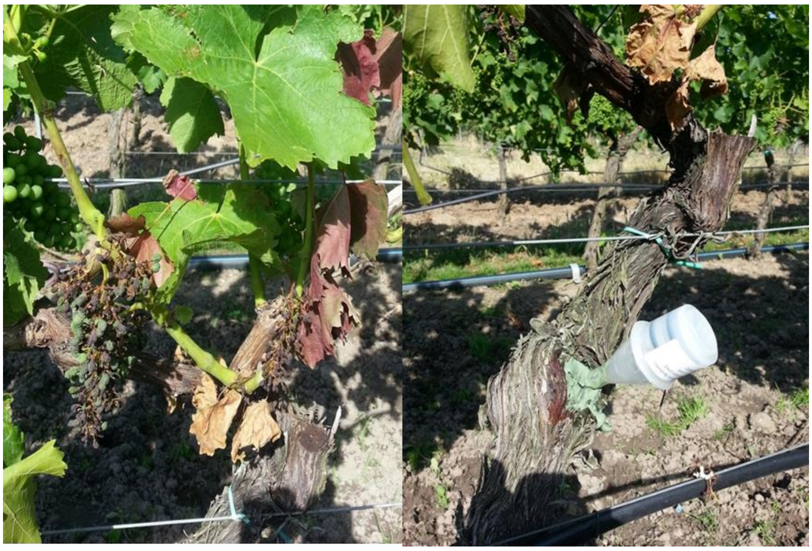

Figure S10. Photographs of the treatment with the nanocarrier dispersion of an Esca-infected grapevine plant (2014, in Forst, Germany).

References

[1] M. Balakshin, E. Capanema, J Wood Chem Technol 2015, 35, 220.

[2] M. Kettering, C. Valdivia, O. Sterner, H. Anke, E. Thines, J Antibiot (Tokyo) 2005, 58, 390.

[3] M. Keller, *The Science of Grapevines*, Academic Press, New York 2010.

[4] K. Rausch, A. Reuter, K. Fischer, M. Schmidt, Biomacromolecules 2010, 11, 2836.
